# Overwintering West Nile virus in active *Culex pipiens* mosquito populations in Greece

**DOI:** 10.1186/s13071-024-06367-6

**Published:** 2024-07-02

**Authors:** Georgios Balatsos, Stavroula Beleri, Nikolaos Tegos, Marina Bisia, Vasileios Karras, Evangelia Zavitsanou, Dimitrios P. Papachristos, Nikos T. Papadopoulos, Antonios Michaelakis, Eleni Patsoula

**Affiliations:** 1 Scientific Directorate of Entomology and Agricultural Zoology, Benaki Phytopathological Institute, 14561 Kifissia, Greece; 2 Department of Public Health Policy, School of Public Health, Division of Infectious, Parasitic Diseases and Zoonoses, University of West Attica, 11521 Athens, Greece; 3 Department of Agriculture, Crop Production and Rural Environment, University of Thessaly, 38446 Magnisias, Greece

**Keywords:** West Nile virus, Overwintering, *Culex pipiens*, Biotypes, Host searching

## Abstract

The flavivirus West Nile Virus (WNV), which is transmitted by mosquitoes, poses a significant threat to both humans and animals, and its outbreaks often challenge public health in Europe and other continents. In recent years, there is an increasing trend of WNV incidence rates across several European countries. However, whether there is a year-round circulation or seasonal introduction has yet to be elucidated. Real-time polymerase chain reaction (PCR) identified WNV-positive *Culex pipiens* mosquitos in 6 out of 146 pools examined in winter 2022 that correspond to three out of the 24 study areas, located in two coastal regions units in Attica, Greece. Spatial dispersion of the six positive pools in the same region suggests a clustered circulation of WNV during the winter of 2022. This is the first study that documents the identification of WNV in *Cx. pipiens* populations, captured in adult traps during winter period. Our findings underscore the need to extend entomological surveillance programs to include the winter period, specifically in temperate climates and historically affected areas by WNV.

**Graphical Abstract:**

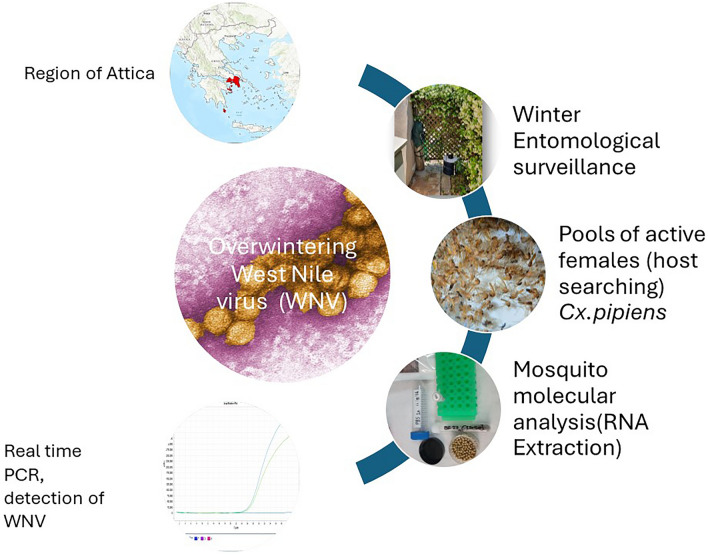

Since its discovery in 1937, West Nile virus (WNV, Flaviviridae), has become one of the most prevalent flaviviruses worldwide, causing severe human diseases in almost all continents [1, 2]. In Europe, the mosquitos *Culex pipiens* and *Culex modestus* are considered the main vectors of WNV, whereas in Greece, the virus has been detected only in *Cx. pipiens* specimens [1, 3–5]. Since the summer of 2010, when the country experienced a major outbreak of WNV infection, annual cases have been consistently recorded across various geographical regions of the country [6]. In 2021, the Hellenic National Public Health Organization (NPHO) reported three cases of WNV among humans in the East Regional Unit of the Attica Region, out of a total of 59 cases recorded across all regions in Greece. However, in the following year, there were no reported cases of WNV in the Attica Region, regardless of the total 286 cases documented country wide [7, 8]. Migrating birds are considered of utmost importance for the introduction of WNV in Greece [7].

Recent entomological surveillance programs in Greece have documented that climate change and particularly warmer winters have increased the duration of seasonal activity for major mosquito species. Notably, the continuous activity of *Cx. pipiens* and the prolonged presence of the Asian tiger mosquito, *Aedes albopictus* have recently been documented in the Attica Region [9–11]. This extension of vectors activity increases the risk of vector-borne diseases transmission and highlights the urgent need for thorough surveillance and intensified mitigation strategies. Nevertheless, whether WNV activity and circulation in vectors and hosts persists year round, has yet to be explored in Greece. The overwintering of WNV in hibernating mosquito vectors has been documented in several countries (e.g., Czech Republic, Germany, Netherlands, and various states in the USA [12–17]. Our study specifically targeted active (host searching) mosquitoes rather than hibernating ones. Therefore, we used adult traps to investigate, for the first time, the possibility of WNV overwintering in active vectors in the Attica Region, aiming to shed light on valuable epidemiological aspects of this disease in Greece.

Although, surveillance programs for mosquitoes in Greece traditionally last from May to November, since 2021 in Attica Region, an all-year-round surveillance has been operating covering more than 55 strategically chosen sites. This expansion of the mosquito population monitoring program, ensures a comprehensive geographical coverage, including sites historically associated with the detection of WNV in humans or vectors. In the present study we considered captures from December to March of 2021–2022 (winter 2022) and 2022–2023 (winter 2023). Within this extensive network, we further elaborated on identifying 24 sampling sites (Fig. 1) where two distinct trapping systems were established: BG Sentinel trap with (1) a BG lure and (2) a BG lure along with carbon dioxide (CO_2_), as attractants. Each trap was positioned on the ground of a shaded, secure, and easily accessible location and operated for 24 h biweekly. The collected mosquitoes were transported to the lab for further analysis using containers containing dry ice [10]. The female mosquitoes were morphologically identified as *Cx. pipiens *sensu stricto and then pooled (2–80 individuals) based on both the date and sampling site. RNA extraction and subsequent TaqMan real-time PCR were performed for the detection of WNV, specifically targeting WNV lineages 1 and 2, nonstructural NS2A region, as previously described [10, 18]. Previous studies in Greece have demonstrated that the main vectors for WNV are the *Cx. pipiens* biotypes, *Cx. pipiens pipiens*, *Cx. pipiens molestus* and the *pipiens*/*molestus* hybrids [4, 5, 10, 19]

**Fig. 1:**
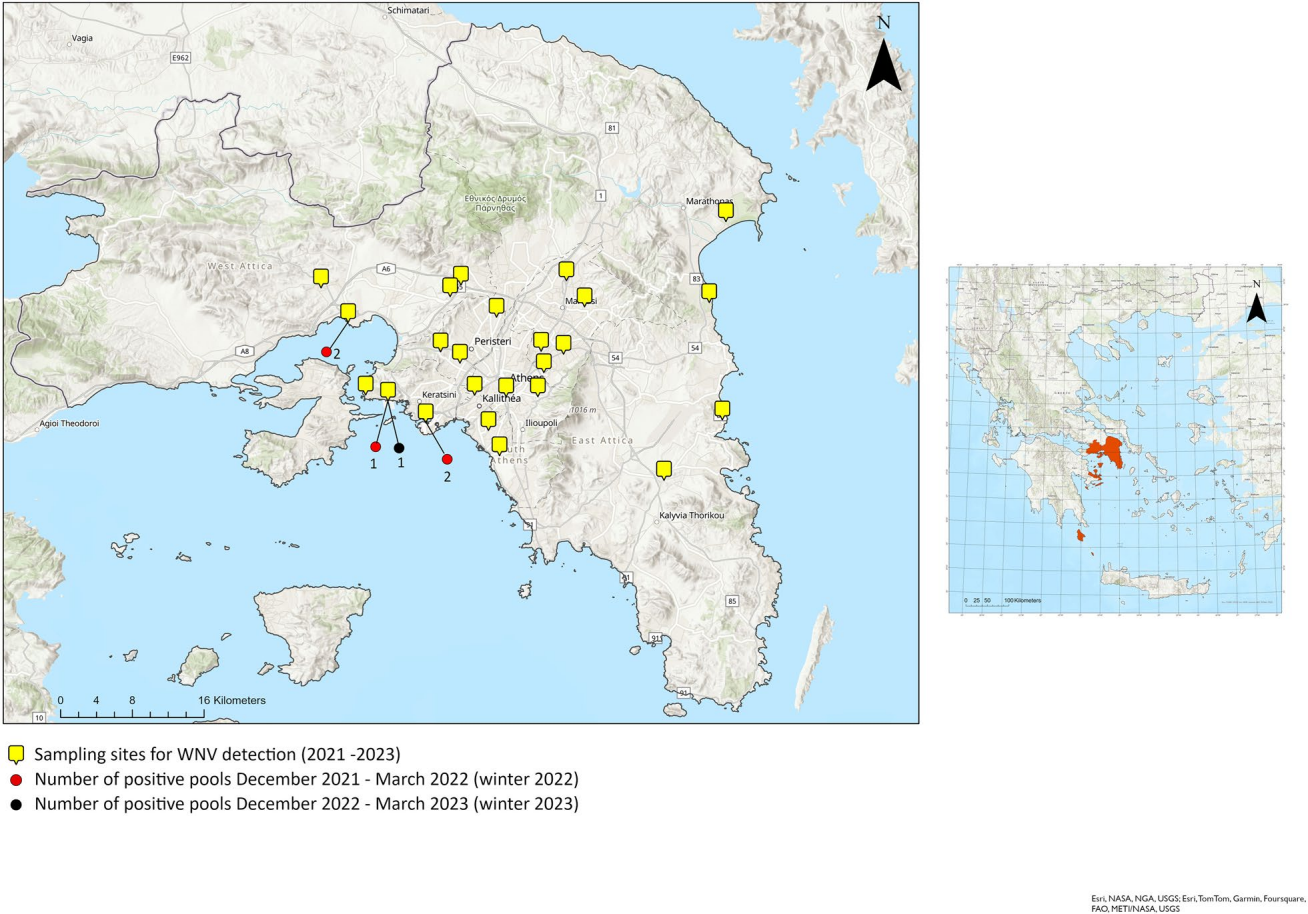
Dispersion of sampling sites (yellow marks) for West Nile virus (WNV) detection in the Attica Region. The red and black dots indicate the positions of the positive samples recorded during winter 2022 and winter 2023, respectively and the frequency of detection

Out of 2264 samples collected in the Attica Region from May 2021 until June 2023, 761 were collected during the winter periods of 2022 and 2023. Mosquitoes’ identification, based on morphological characters, was performed after careful examination under a NIKON SMZ645 Stereo Microscope (Nikon Instruments Inc., Surrey, UK), using appropriate dichotomous keys [20–22]. Because the biotypes are indistinguishable morphologically, adult females were characterized morphologically as *Cx. pipiens *sensu stricto and then stored in −80 °C for further processing regarding WNV detection [20]. The summary of the relative abundance of each mosquito species collected during the winter periods presented in Table 1 highlights the *Cx. pipiens* as the predominant species.

**Table 1:** Relative abundance of female mosquito species collected during the winter periods, from December to March of 2021–2022 (winter 2022) and from December to March of 2022–2023 (winter 2023)

Month, year	Mosquito species				Total number of species per month	Relative abundance for *Cx. pipiens* (%)
	*Culex pipiens*	*Aedes albopictus*	*Culiseta longiareolata*	*Aedes detritus*		
December, 2021	112	1	1	1	115	97.4
January, 2022	89	0	2	0	91	97.8
February, 2022	28	0	5	0	33	84.8
March, 2022	63	0	7	0	70	90.0
December, 2022	800	22	7	0	829	96.5
January, 2023	404	1	3	0	408	99.0
February, 2023	49	0	4	0	53	92.5
March, 2023	105	0	7	0	112	93.8

Out of 19,176 females (1285 pools), *Cx. pipiens* mosquitoes, 1650 (225 pools) were captured during both winter periods. In more details, a total of 292 (79 pools) female *Cx. pipiens* were captured in winter 2022, and 1358 (146 pools) were captured in winter 2023. The WNV was detected only in winter 2022 in 6 out of the 225 tested pools (4.1% positive pools; Table 2; Fig. 1). All positive samples related to WNV overwintering were detected in coastal areas of Piraeus (main port of Greece) and West Attica (urban and industrial zones) regional units that are located next to each other, regardless of the presence of CO_2_ in the traps.

**Table 2:** Information regarding the pools positive for West Nile virus (WNV) was obtained using two types of BG sentinel 2 (BG) traps. Each trap was operated for 24 h biweekly, and two distinct trapping systems were established: BG Sentinel trap with (1) a BG lure and (2) a BG lure along with carbon dioxide (CO_2_), as attractants

Municipality (Regional unit)	GPS (BG sites)	Trap type	Sampling date	No. of female* Cx. pipiens*
Elefsina (West Attica)	38.038896°, 23.535081°	BG, BG Lure + CO_2_	5 January 2022	10
Piraeus (Piraeus)	37.938403°, 23.634192°	BG, BG Lure	8 January 2022	2
Piraeus (Piraeus)	37.938403°, 23.634192°	BG, BG Lure	11 February 2022	2
Perama (Piraeus)	37.960037°, 23.586211°	BG, BG Lure	12 February 2022	1
Elefsina (West Attica)	38.038896°, 23.535081°	BG, BG Lure + CO_2_	30 March 2022	9
Perama (Piraeus)	37.960037°, 23.586211°	BG, BG Lure + CO_2_	1 December 2022	6

## Discussion

The thorough and comprehensive mosquito sampling plan implemented in Attica Region, revealed, for the first time, the overwintering of WNV in active vectors captured in adult mosquito traps. In contrast with other studies worldwide focused on the detection of WNV in hibernating mosquitoes, our research highlights the outdoor activity of *Cx. pipiens* and the occurrence of WNV during the winter months. Since the persistence of the virus in nature in hibernating vectors has been demonstrated to play an important role for the maintenance of the virus from year to year, the presence in active vectors should also be critical for both maintenance/circulation and proliferation that is of utmost importance from an epidemiological point of view [14]. By demonstrating the outdoor activity of *Cx. pipiens* and the presence of WNV during winter, our study highlights the need for broader surveillance strategies to capture the full spectrum of vector activity and disease transmission potential, particularly in regions experiencing milder winter climates.

In our study, positive pools for WNV were detected at 3 out of 24 spots located within two coastal regional units of Attica, suggesting the occurrence of localized circulation of WNV during the winter of 2022. As a limitation of the current study we consider the lack of identifying the *Cx. pipiens* specimens to biotypes level, because pools of adults and not individuals were used to detect the WNV. This approach has been selected due to limited resources. Nevertheless, our findings are raising inquiries about the ecological and environmental factors driving the dynamics of WNV transmission in localized areas of the Mediterranean coast. While wetlands and bird migration routes have traditionally been associated with WNV transmission both to humans and vectors, our study reveals a potential link between urban and industrial zones, port areas, and WNV circulation in vectors [9, 23]. Furthermore, the inclusion or exclusion of CO_2_ as an attractant in traps has not impact on the detection of WNV during winter surveillance activities (Table 2). Potential alternative factors should be considered for further analysis including temperature fluctuations, humidity levels, and specific environmental cues that influence mosquito behavior and the virus transmission during winter period [24].

## Conclusions

The epidemiological importance of our findings is reflected in the implementation of a vector surveillance program conducted annually, aiming to serve as a crucial component in monitoring both vectorial and WNV dynamics in an area. These findings underscore the importance of refining and optimizing surveillance methodologies to enhance efficiency and resource utilization in our ongoing efforts to monitor and understand the dynamics of WNV circulation and potential transmission during the winter period. This highlights the necessity of year-round surveillance to effectively monitor and mitigate the spread of WNV. Recommendations advocate the year-round extension of all surveillance programs in Greece and ideally to other Mediterranean countries, to include the winter period, especially in areas where WNV outbreaks have been historically recorded and those affected by climate change. By targeting these regions, we can better understand the evolving transmission dynamics of WNV and implement preventive measures to limit its spread. This proactive approach is crucial for safeguarding public health and minimizing the impact of vector-borne diseases.

## Data Availability

All the data are available in the manuscript.
